# Understanding risk factors for low anterior resection syndrome in a South American cohort

**DOI:** 10.1590/0102-67202025000037e1906

**Published:** 2025-10-27

**Authors:** María Inés GAETE, Cristián Ignacio JARRY, Daniel MORENO, José Tomás LARACH, Felipe BELLOLIO

**Affiliations:** 1 Pontificia Universidad Católica de Chile, Department of Digestive Surgery, Coloproctology Unit – Santiago, Metropolitana, Chile.

**Keywords:** Rectal Neoplasms, Low Anterior Resection Syndrome, Quality of Life, Cohort Studies, Risk Factors, Neoplasias Retais, Síndrome de Ressecção Anterior Baixa, Qualidade de Vida, Estudos de Coortes, Fatores de Risco

## Abstract

**Background:**

Low Anterior Resection Syndrome (LARS) is a common postoperative bowel dysfunction in patients undergoing sphincter-preserving surgery for rectal cancer. Symptoms include fecal and gas incontinence, urgency, increased bowel frequency, and fragmented evacuations. LARS significantly impairs quality of life, affecting up to 90% of patients. Various factors contribute to its development, such as tumor height, extent of mesorectal excision, preoperative radiotherapy, and ileostomy. However, these factors are less studied in South American populations, where racial, cultural, and healthcare system differences may influence outcomes.

**Aims:**

The aim of the study was to evaluate risk factors associated with LARS in a Chilean cohort of rectal cancer patients, with emphasis on cases classified as severe.

**Methods:**

A non-concurrent prospective cohort study including patients who underwent low anterior resection between 2012 and 2021. Perioperative data collected included tumor height, surgical procedure type, preoperative radiotherapy, and protective ileostomy. Univariate and multivariate analyses were conducted to identify factors significantly associated with severe LARS, using the LARS score adapted to Chilean Spanish.

**Results:**

A total of 110 patients were included, with a median follow-up of 51 months. LARS was identified in 52.7% of cases, with 29.1% classified as major. Younger age, lower tumors, total mesorectal excision, preoperative radiotherapy, and ileostomy were significantly associated with severe LARS in univariate analysis. In multivariate analysis, only younger age and preoperative radiotherapy remained as independent risk factors.

**Conclusions:**

In this Chilean cohort, nearly half of patients undergoing sphincterpreserving surgery for rectal cancer developed LARS. About one-third had the severe form, highlighting the need for targeted strategies to mitigate LARS and improve patient quality of life.

## ARTICLE HIGHLIGHTS

LARS significantly impairs quality of life, highlighting the need for personalized perioperative strategies.

Nearly half of patients developed LARS, with 29.1% classified as major LARS after rectal cancer surgery.

Preoperative radiotherapy and younger age were independent predictors of severe LARS.

## Summary

**CENTRAL MESSAGE:**

Low Anterior Resection Syndrome (LARS) is a recognized complication following sphincter-sparing rectal cancer surgery and is associated with considerable impairment in bowel function. The symptoms vary widely among patients and may include fecal and gas incontinence, urgency, increased frequency of defecation, and stool clustering. Such disturbances can lead to relevant limitations in daily activities, with up to 90% of patients reporting a significant impact on their quality of life (QoL). The pathophysiology of LARS is multifactorial, with several key risk factors contributing to its development.

**PERSPECTIVES:**

This study underscores the significant burden of Low Anterior Resection Syndrome (LARS) among Chilean patients undergoing sphincter-sparing rectal cancer surgery, with half of them experiencing some degree of LARS and nearly one-third classified as having major LARS. Among the evaluated risk factors, preoperative radiotherapy and younger age emerged as independent predictors of major LARS, while total mesorectal excision and protective ileostomy were also associated with increased severity, though they did not reach statistical significance in the multivariate analysis. These findings emphasize the critical role of comprehensive preoperative counseling and multidisciplinary decision-making in mitigating LARS risk and optimizing long-term functional outcomes in rectal cancer patients.

## VISUAL ABSTRACT

**Figure:**
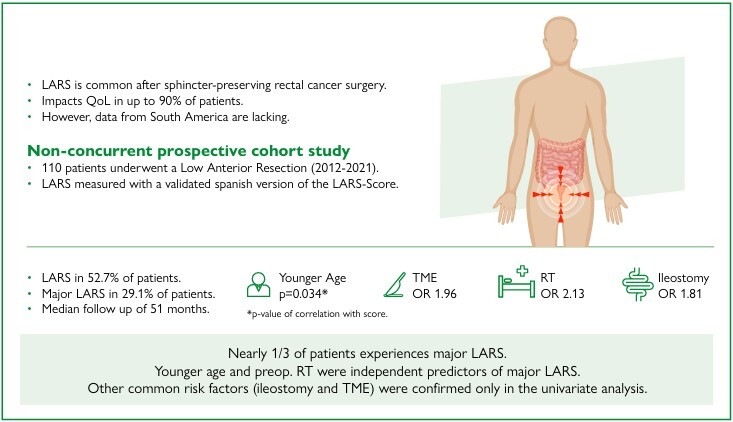

## RESUMO

**Racional:**

A Síndrome da Resseção Anterior Baixa (SRAB) é uma disfunção intestinal comum após cirurgia para câncer retal com preservação do esfíncter. Os sintomas incluem incontinência fecal e de gases, urgência evacuatória, evacuações frequentes e fragmentadas. A SRAB compromete a qualidade de vida em até 90% dos casos. Diversos fatores, como altura tumoral, extensão da excisão mesorretal, radioterapia pré-operatória e ileostomia de proteção, estão associados ao seu desenvolvimento. Contudo, estes fatores são menos estudados em populações sul-americanas, onde diferenças culturais, raciais e nos sistemas de saúde podem influenciar os resultados.

**Objetivos:**

Avaliar os fatores de risco associados ao SRAB numa coorte chilena com câncer retal, com foco nos casos classificados como SRAB grave.

**Métodos:**

Um estudo de coorte prospectivo não concorrente com pacientes submetidos à resseção anterior baixa entre 2012 e 2021. Foram coletados dados como altura tumoral, tipo de cirurgia, radioterapia pré-operatória e ileostomia de proteção. Análises univariada e multivariada foram realizadas utilizando o "SRAB score" adaptado ao espanhol chileno.

**Resultados:**

Foram incluídos 110 pacientes, com seguimento mediano de 51 meses. SRAB foi identificado em 52,7% dos casos, sendo 29,1% classificados como graves. Idade mais jovem, tumores mais baixos, excisão total do mesorreto, radioterapia pré-operatória e ileostomia de proteção associaram-se significativamente ao SRAB grave. Na análise multivariada, apenas idade mais jovem e radioterapia mantiveram-se como fatores independentes.

**Conclusões:**

Quase metade dos pacientes submetidos à cirurgia de preservação do esfíncter para câncer retal desenvolveu SRAB, nesta coorte chilena. E cerca de um terço apresentou a forma grave, destacando a necessidade de estratégias direcionadas para mitigar a SRAB e melhorar a qualidade de vida dos pacientes.

## INTRODUCTION

 Low Anterior Resection Syndrome (LARS) is a recognized complication following sphincter-sparing rectal cancer surgery and is associated with considerable impairment in bowel function^3,14,15,20^. The symptoms vary widely among patients and may include fecal and gas incontinence, urgency, increased frequency of defecation, and stool clustering. Such disturbances in bowel function can lead to substantial limitations in daily activities, with up to 90% of patients reporting a significant impact on their quality of life (QoL)^3,8,11,13-15,18-21^. The pathophysiology of LARS is multifactorial, with several key risk factors contributing to its development. Tumor height and the extent of mesorectal excision play critical roles, with lower tumors and those requiring total mesorectal excision (TME) being more frequently associated with severe symptoms. The mechanism underlying this association is likely related to the increased risk of nerve damage in patients with lower tumors undergoing TME. Preoperative radiotherapy is another well-established risk factor for LARS, as it is associated with radiation-induced damage to the rectal wall, leading to structural and functional changes. In addition, the use of a protective ileostomy, while beneficial in reducing the risk of anastomotic leakage, has been implicated as a contributing factor to the development of LARS, likely due to delayed bowel function recovery and altered gut microbiota^4,5,17,22,23,25,26^. 

 Despite the well-established impact of LARS on patient outcomes, its diagnosis and classification have not always been standardized^1,3,12,14,15^. Among a variety of definitions and tools, the LARS Score has emerged as a validated tool for characterizing the severity of symptoms^6^. The score has been widely adopted for both clinical and research purposes due to its strong correlation with patient-reported quality of life^6,13,20^. Efforts have also been made to develop predictive models for LARS. One such model, the Pre-Operative LARS Score (POLARS), integrates clinical variables such as age, tumor height, extent of mesorectal excision, receipt of preoperative radiotherapy, and presence of a protective ileostomy to predict postoperative bowel dysfunction^2^. However, while POLARS has been validated in European cohorts, its applicability to Latin American populations remains uncertain. A recent study in Chilean patients undergoing rectal cancer surgery demonstrated poor agreement between POLARS and LARS scores, raising concerns about the external validity of the predictive model^9^. 

 Given the potential differences in risk factor interactions and the impact of LARS across diverse populations, there is a critical need to explore these variables within Latin American settings. Additionally, evolving oncological paradigms suggest that in selected cases, preoperative radiotherapy and ileostomy may be avoidable, highlighting the importance of reassessing their risks and benefits in different populations^10^. To address this gap, this study aims to explore and validate traditional risk factors for LARS within a Chilean cohort and to identify those most strongly associated with major LARS. This emerges as a relevant step toward addressing risk stratification strategies and optimizing perioperative decision-making within our population. 

## METHODS

### Study design and participants

 A non-concurrent prospective cohort study was conducted at a high-volume academic hospital. Patients diagnosed with rectal cancer who underwent curative-intent surgery between 2012 and 2021 were included based on the following eligibility criteria:

1. Patients aged 18 years or older,
2. Underwent low anterior resection with anastomosis,
3. Had a tumor located within 15 cm of the anal margin and
4. Were available for telephone follow-up.

Patients were excluded if they had undergone surgical procedures other than TME or partial mesorectal excision (PME), had incomplete perioperative data, or had conditions precluding reliable data collection. All cases were reviewed in a multidisciplinary oncologic board, which determined the indication for neoadjuvant therapy and the surgical approach. Protective ileostomy was routinely performed in patients receiving preoperative radiotherapy or requiring TME, whereas for other patients, it was left to the discretion of the operating surgeon. 

#### Data collection and low anterior resection syndrome assessment

 A standardized data collection protocol was implemented to record demographic and perioperative variables. The primary outcome was the presence and severity of LARS, evaluated using the LARS Score previously adapted for Chilean Spanish^16^. Patient follow-up was conducted through telephone interviews, during which trained researchers administered the LARS questionnaire. The responses were used to classify patients into three predefined categories: No LARS (<21 points); Minor LARS (21–29 points); Major LARS (≥30 points). 

#### Statistical analysis

 Descriptive statistics were employed to summarize the demographic and clinical characteristics of the study population. Continuous variables were presented as mean (standard deviation [SD]) or median [interquartile range], as appropriate. The normality of distributions was assessed using the Shapiro-Wilk test, and non-parametric tests were used as assumptions of normality were not confirmed among most variables. Comparisons between categorical variables were performed using the Fisher’s exact test, while differences in continuous variables were assessed using the Mann-Whitney U test. Spearman’s correlation coefficient was employed to evaluate the association between continuous variables and LARS scores. To identify independent predictors of major LARS, a binary logistic regression model was fitted. Variables with a p-value <0.1 in the univariate analysis were included in the final multivariate model. The results were expressed as odds ratios (ORs) with 95% confidence intervals (CIs). All statistical analyses were conducted using SPSS® version 28 (IBM®). A p-value <0.05 was considered statistically significant. 

### Compliance with ethical standards

 The study protocol was approved by the Scientific Ethics Committee of the Faculty of Medicine at Pontificia Universidad Católica de Chile (ID: 220106001). All patients provided informed consent for participation and data usage. 

## RESULTS

### Patient characteristics and low anterior resection syndrome incidence

 A total of 110 patients were included in the study. The mean age of the cohort was 59.36 years (SD 11.78), and 55% were female. The median follow-up time was 51 months (25–76 months). Regarding surgical procedures, TME was performed in 60% of patients, while PME was performed in 40%. Patients who underwent TME had significantly lower median tumor height (8 [6–12] cm) compared to those who underwent PME (14.5 [9.25–15] cm, p<0.001). Preoperative radiotherapy was administered to 41% of patients, and a protective ileostomy was performed in 55% of cases (Table 1). 

**Table 1:** Demographic characteristics and low anterior resection syndrome incidence.

Variables		Included patients (n=110)
Age (years), mean (SD)		59.36 (11.78)
Female sex, n (%)		61 (55.5)
Time to survey administration (months), median (IQR)		51 [25–76]
Type of surgery, n (%)		
	TME	66 (60)
	PME	44 (40)
Tumor height (cm), median (IQR)		
	TME	8 [6–12]
	PME	14.5 [9.25–15]
Preoperative radiotherapy, n (%)		45 (41)
Protective ileostomy, n (%)		61 (55.5)
LARS incidence, n (%)		
	No LARS	52 (47.3)
	Minor LARS	26 (23.6)
	Major LARS	32 (29.1)

SD: standard deviation; IQR: interquartile range (reported as p25–p75); TME: total mesorectal excision; PME: partial mesorectal excision; LARS: low anterior resection syndrome.

 At follow-up, 52.7% of patients exhibited some degree of LARS, with 23.6% classified as minor LARS and 29.1% classified as major LARS (Table 1). 

### Univariate analysis of risk factors for low anterior resection syndrome

**Figure 1:**
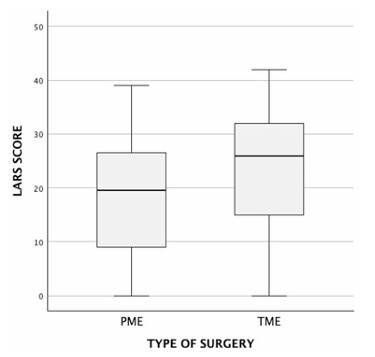
Low anterior resection syndrome score by type of surgery. Partial mesorectal excision versus total mesorectal excision. LARS: low anterior resection syndrome; PME: partial mesorectal excision; TME: total mesorectal excision.

**Figure 2:**
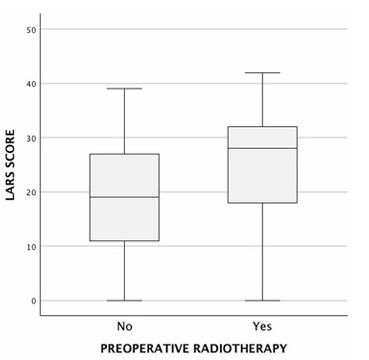
Low anterior resection syndrome score by history of preoperative radiotherapy. LARS: low anterior resection syndrome.

**Figure 3:**
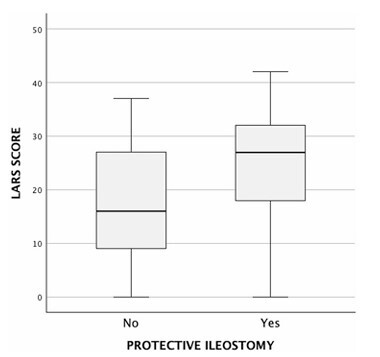
Low anterior resection syndrome score by protective ileostomy status after surgery. LARS: low anterior resection syndrome.

 The univariate analysis revealed several factors significantly associated with LARS. Tumor height was inversely correlated with LARS severity (p=-0.269, p=0.004, p<0.05), indicating that lower tumors were associated with a higher reported LARS score. The surgical approach also influenced LARS outcomes. Patients undergoing TME had significantly higher scores compared to those who underwent PME (median LARS score: 26 [14.5–32] vs. 19.5 [9–16.75], p=0.008, p<0.05) (Figure 1). Similarly, patients who received preoperative radiotherapy had significantly higher LARS scores compared to those who did not (median score: 28 [18–32] vs. 19 [10–27], p<0.001) (Figure 2). The presence of a protective ileostomy was also associated with higher LARS scores, with patients who had an ileostomy exhibiting a median score of 27 [18–32], compared to 16 [9–27] in those without an ileostomy (p<0.001) (Figure 3). Finally, younger age was associated with higher LARS scores, as evidenced by a negative correlation (p=-0.234, p=0.014, p<0.05). Anastomotic leak was not significantly associated with increased LARS severity in this cohort. 

### Sub-analysis of major low anterior resection syndrome

 Patients with major LARS had a significantly lower median tumor height compared to those without LARS or with minor LARS (8 [6–12] cm vs. 12 [7–15] cm, p=0.047, p<0.05). Additionally, patients who underwent TME had 1.96 times the odds of developing major LARS compared to those who underwent a PME (OR 1.96; 95%CI 1.12–1.96, p=0.01, p<0.05). Also, patients who received preoperative radiotherapy had 2.13 times the odds of developing major LARS compared to those who did not (OR 2.13; 95%CI 1.41–3.24, p<0.001). Finally, the presence of a protective ileostomy was associated with an OR of 1.81 for developing major LARS compared to patients without an ileostomy (OR 1.81; 95%CI 1.32–2.43, p<0.001) (Table 2). 

**Table 2:** Demographic characteristics and low anterior resection syndrome incidence.

Variable/Category		Univariate analysis			Multivariate analysis		
		OR*	95%CI	p-value	OR	95%CI	p-value
Age		-0.202	—	0.034	0.96	[0.95–0.99]	0.041
Tumor height		-0.269		0.004	1.16	[0.97–1.38]	0.103
Sex						
	Male	1.14	[0.50–2.61]	0.753			
	Female	Reference					
Surgery						
	TME	1.96	[1.12–1.96]	0.010	2.61	[0.83–8.18]	0.099
	PME	Reference			Reference		
Preoperative radiotherapy						
	Yes	2.13	[1.41–3.24]	0.001	3.95	[1.03–15.19]	0.045
	No	Reference			Reference		
Protective ileostomy						
	Yes	1.81	[1.32–2.43]	0.001	3.52	[0.95–13.07]	0.060
	No	Reference			Reference		

OR: odds ratio for categorical variables; CI: confidence interval; TME: total mesorectal excision; PME: partial mesorectal excision

Spearman’s Rho *for numeric variables.

### Multivariate analysis: independent predictors of major low anterior resection syndrome

 A multivariate logistic regression model was built to assess the independent contributions of the significant factors identified in the univariate analysis of major LARS development. After adjusting for confounding variables, preoperative radiotherapy (OR 3.95, 95%CI 1.03–15.19, p=0.045, p<0.05) and younger age (OR 0.96, 95%CI 0.95–13.06, p=0.041, p<0.05) emerged as independent predictors of major LARS. Although TME (OR 2.61, 95%CI 0.83–8.18, p=0.099, p>0.05) and protective ileostomy (OR 3.52, 95%CI 0.95–13.07, p=0.060, p>0.05) did not reach statistical significance, their ORs suggest potential clinical relevance, indicating a meaningful association with major LARS. Tumor height was not found to be a statistically significant predictor in the multivariate model. 

## DISCUSSION

 This study examined a cohort of patients undergoing sphincter-sparing rectal cancer surgery at a national university center, revealing that 52.7% of patients developed some degree of LARS, with 29.1% classified as major LARS, based on a median follow-up of 51 months. Our findings confirm that tumor height, TME, preoperative radiotherapy, protective ileostomy, and younger age are associated with increased LARS scores. When specifically evaluating major LARS, these risk factors remained significant, with preoperative radiotherapy, TME, and ileostomy demonstrating ORs close to 2. The multivariate analysis showed that younger age and preoperative radiotherapy emerged as independent risk factors, while TME and protective ileostomy maintained OR magnitudes that suggest clinical relevance, despite not reaching statistical significance. 

 The prevalence of major LARS in our cohort (29.1%) was slightly lower than the 40–50% reported in meta-analyses and large series^4,1,20,23^. Regarding the identified risk factors, relatively few studies have reported them as ORs, making direct comparisons challenging. A study involving 129 patients with a 3.17-year follow-up found an OR of 2.9 for ileostomy and 6.55 for radiotherapy, values notably higher than those observed in our cohort^5^. Similarly, a multivariate analysis conducted by Emmertsen et al.^7^, which included sex, surgical technique (TME vs. PME), radiotherapy, anastomotic leakage, and neorectal reservoir configuration at 12 months, found that preoperative radiotherapy (OR 2.41) and TME (OR 2.81) were the only significant predictors of major LARS. Consistent with our findings, neither anastomotic leakage nor sex was a significant risk factor in their study. 

 Several systematic reviews and meta-analyses have investigated risk factors for LARS. Notably, only one study from South America (Brazil) was identified, which reported female sex, chemoradiotherapy, and ileostomy as risk factors^17^. However, this study lacked a validated methodology for systematically diagnosing LARS, limiting the generalizability of its findings. In contrast, a meta-analysis by Ye et al.^25^, predominantly based on Chinese studies, identified neoadjuvant therapy, anastomotic leakage, anastomosis <5 cm from the anal verge, and a stoma as predictors of LARS. Another systematic review by Vogel et al.^24^, which included nine studies using various LARS assessment tools, reported a combined OR of 2.84 for ileostomy. This suggests that while ileostomy remains a significant risk factor, the magnitude of its effect may vary across populations and study methodologies. 

### Strengths and limitations

 Several limitations should be acknowledged. First, we did not include additional diagnostic or therapeutic assessments during the perioperative period, which could have provided a more comprehensive evaluation of functional outcomes. Although the LARS Score is a validated tool that correlates well with quality of life, other QoL scales were not included in this study. Furthermore, postoperative functional anorectal assessments were not recorded, limiting our ability to correlate physiological parameters with LARS severity. 

 Another important limitation is that interventions for LARS management and prevention, such as pelvic floor rehabilitation, pharmacological therapy, and dietary modifications, were not systematically evaluated, despite the likelihood that a proportion of patients were receiving them. Additionally, the sample size may have limited the statistical power to detect significant associations for ileostomy and TME in the multivariate analysis, despite their clinical relevance. A larger cohort with prolonged follow-up could provide further insights and allow for clinically relevant estimates such as the number needed to treat or harm, as well as facilitate the development of predictive models tailored to this population. 

### Clinical implications and future directions

 This study provides essential epidemiological data on the incidence of LARS in a Chilean population, laying the groundwork for strategies aimed at reducing its functional impact. Among these, the selective avoidance of preoperative radiotherapy and ileostomy in carefully chosen patients may help minimize functional sequelae. However, such decisions must be based on high-quality evidence, considering both patient preferences and oncological outcomes. 

 The significant burden of LARS, particularly major LARS in 29% of patients, emphasizes the need for multidisciplinary teams focused on improving QoL. Incorporating specialized rehabilitation programs, psychological support, and personalized postoperative management may facilitate optimal recovery and enhance social and occupational reintegration. Finally, the centralization of complex pelvic oncologic care in high-volume centers should be considered, as it may improve outcomes through greater expertise in functional preservation strategies and perioperative optimization. 

## CONCLUSIONS

 This study underscores the significant burden of LARS among Chilean patients undergoing rectal cancer surgery, with half of them experiencing some degree of LARS and nearly one-third classified as having major LARS. Among the evaluated risk factors, preoperative radiotherapy and younger age emerged as independent predictors of major LARS, while TME and protective ileostomy were also associated with increased severity, though they did not reach statistical significance in the multivariate analysis. These findings emphasize the critical role of comprehensive preoperative counseling and multidisciplinary decision-making in mitigating LARS risk and optimizing long-term functional outcomes in rectal cancer patients. Moreover, the results highlight the necessity for further research to develop refined risk stratification models and tailored postoperative rehabilitation strategies that could help address functional impairments across diverse populations. Given that existing predictive tools have primarily been validated in different racial, cultural, and healthcare contexts, a need exists to adapt and validate these models to improve clinical applicability and personalized patient care in South American populations. 

## Data Availability

The information regarding the investigation, methodology, and data analysis of the article is archived under the responsibility of the author.
